# Chemical profiles of the active fraction from *Prinsepia utilis* Royle leaves and its anti-benign prostatic hyperplasia evaluation in animal models

**DOI:** 10.1186/s12906-021-03446-4

**Published:** 2021-10-29

**Authors:** Ying Peng, Chongsheng Peng, Yang Wu, Chongzhi Sun, Xiaobo Li

**Affiliations:** School of Pharmacy, Shanghai Jiao Tong University, No. 800 Dongchuan Road, Minhang District, Shanghai, 200240 People’s Republic of China

**Keywords:** *Prinsepia utilis* Royle, Flavonoid, Benign prostatic hyperplasia, UPLC-QTOF-MS, Quantification

## Abstract

**Background:**

The *Prinsepia utilis* Royle leaves (*P. utilis*) is a folk herb used for benign prostatic hyperplasia (BPH) control by ethnic minorities for centuries in China with rich in resources. Our previous studies have confirmed the anti-BPH effect of its water extract (QCJ) and the active fraction (Fr. B) separated from the QCJ by animal test. The Fr. B from *P. utilis* should be a potential candidate for BPH control.

**Methods:**

In this study, the chemical ingredients of Fr. B were identified by UPLC-QTOF-MS, and quantified by HPLC. Murine animal models were divided into 8 groups, Sham rats, BPH rats, BPH rats administered with finasteride (1 mg/kg), BPH rats administered with Pule’an (460 mg/kg), BPH rats administered with low, high dosage of QCJ (860 mg/kg, 2580 mg/kg respectively), BPH rats administered with low, high dosage of Fr. B (160 mg/kg, 480 mg/kg respectively). The expression of vascular endothelial growth factor (VEGF) in the prostate tissue of rats was tested, and serum levels of dihydrotestosterone (DHT), testosterone (T), estradiol (E2), interleukin-6 (IL-6), tumor necrosis factor-α (TNF-α) and total superoxide dismutase (SOD), glutathione peroxidase (GSH-Px), catalase (CAT), malondialdehyde (MDA) in prostate homogenate were measured. One-way ANOVA followed by LSD was used for statistical analysis.

**Results:**

The BPH rats treated by Fr. B exhibited significant reductions of VEGF and MDA levels, as well as significant increases of SOD, GSH-Px and CAT in the prostate tissue after 28 day administration (*P* < 0.05). Moreover, Fr. B significantly reduced DHT, DHT/E2 ratio, TNF-α, while increased T levels in serum of BPH rats (*P* < 0.05). UPLC-QTOF-MS analysis revealed 10 flavonoids as the key constituents of this fraction, which accounted for 54.96% of all substance of Fr. B. The relative contents of compound 1, 2 are 11.1%, 13% in Fr. B respectively.

**Conclusions:**

These results indicated that the Fr. B obtained from *P. utilis* alleviated the symptoms of BPH rats through multiple mechanisms including reduction of DHT/E2 ratio, inhibition of growth factor, anti-inflammation and anti-oxidation, in which flavonoids might be the key constituents. It supported the hypothesis that the Fr. B should be further explored as a candidate for BPH patients.

## Background

*Prinsepia utilis* Royle (*P. utilis*) is belonged to prinsepia genus (rosaceae), mainly distributed in southwest China such as Yunnan and Guizhou province. It is a food medicine homologous plant, of which the roots, leaves and fruits are traditionally used to treat toothache, carbuncle and gangrene [1]. Pharmacological studies showed that the stems and leaves of *P. utilis* had the functions of anti-osteoporosis [2], regulating blood lipids [3], anti-tumor [4], anti-oxidation [2], anti-inflammation [5], anti-bacteria [6], etc. Interestingly, as a multi-ethnic herb, the leaves of *P. utilis* is used as tea in folk for benign prostatic hyperplasia (BPH) symptom control by Naxi, Bai, Yi, Mosuo and other ethnic minorities for centuries in Yunan province.

Generally, BPH is considered to be a product of androgen action upon an aging prostate. But longitudinal epidemiological studies showed that androgen levels were unlikely to be solely responsible [7]. There are many potential etiological factors contributing to BPH pathogenesis such as inflammation, oxidative stress, imbalance between prostate cell growth and apoptosis [8, 9]. Our previous studies confirmed the anti-BPH activities of *P. utilis* leaves extract (QCJ) on BPH rats and the Fr. B separated from QCJ as the active fraction [10]. However, whether its anti-BPH activity is related to anti-inflammation, anti-oxidation or anti-androgenic need further study.

According to the chemical reports on *P. utilis*, flavonoids, amino acids, steroids and terpenoids have been isolated from *P. utilis* fruit, stems and leaves [11]. The content of total flavonoids is up to be 7.25% in *P. utilis* fruit [12], 3% in stems and leaves of *P. utilis* [13]. The flavonoids usually exhibited potent anti-inflammatory and antioxidant properties. It was reported that flavonoid-rich fraction from *P. utilis* fruits exhibited strong radical scavenging activities [14]. Many medicinal plants rich in flavonoids showed well anti-BPH potential [15, 16], which were reported to exert BPH protective effect through regulating inflammatory responses and reducing oxidative stress.

Therefore, these findings led us to further analyze the chemical ingredients of Fr. B with UPLC-QTOF-MS, assess its therapeutic effects on testosterone-induced BPH rats by oral administration of Fr. B and compared with finasteride and Pule’an used as first-line therapy in China in terms of its efficacy and possible mechanisms as anti-androgenic, anti-oxidant and anti-inflammatory in this study.

## Materials and methods

### Extraction and isolation of Fr. B

*P. utilis* leaves was collected from Dali, Yunnan, China and authenticated by one of the authors Xiaobo Li. Voucher specimen has been deposited at herbarium of School of Pharmacy, Shanghai Jiao Tong University, Shanghai, China. Preparation of Fr. B was previously described by Wu et al [10]. Dried and powdered leaves (2 kg) were re-fluxed 3 times with distilled water. The combined extraction was filtrated and then concentrated under reduced pressure to obtain a crude aqueous extract (QCJ). The sample was redissolved, and then separated by macroporous resin AB-8. After adsorption, the resin was washed with 5% ethanol without collection, followed by 40% ethanol, the elution was then evaporated under vacuum at 65 °C and lyophilized to obtain Fr. B.

### UPLC-QTOF-MS analysis of Fr. B

The qualitative chemical profiles of Fr. B was analyzed by UPLC-QTOF-MS in MS^E^ mode which was performed on a Waters ACQUITY UPLC I-Class system (Waters Corp., Milford, MA, United States) with an ACQUITY UPLC BEH C18 column (100 × 2.1 mm, 1.7 μm, Waters Corp., United States) by gradient elution using 0.1% formic acid in water (A) and 0.1% formic acid in acetonitrile (B) at a flow rate of 0.4 ml/min. The gradient profile was 0–2 min (A: 90%), 2–9 min (A: 90–80%), 9–11 min (A: 80–65%), 11–14 min (A: 65–0%). The injection volume was 1 μl. The temperature of the column oven was set to 45 °C. Mass spectrometry was carried out using a Waters VION IMS QTOF mass spectrometer (Waters Corp., Milford, MA, United States). Ionization was performed in both positive and negative electrospray ionization (ESI) mode. The MS parameters were as follows, capillary voltage, 2.5 kV; cone voltage, 40 V; source temperature, 115 °C; desolvation temperature, 450 °C; gas flows of cone and desolvation, 50 and 900 l/h. A MSE (Mass Spectrometry Elevated Energy) experiment in two scan functions was carried out as follows. Function 1 (low energy), m/z 50–1000, 0.2 s scan time, 0.02 s inter-scan delay, 4 eV collision energy. Function 2 (high energy), m/z 50–1000, 0.2 s scan time, 0.02 s inter-scan delay, collision energy ramp of 20–45 eV. The data were processed using UNIFI 1.8.1 software (Waters Corp., Milford, MA, United States).

### Quantitative analysis of Fr. B

The quantitative analysis of Fr. B were performed on an Agilent 1200 HPLC (high-performance liquid chromatography) system, equipped with a quaternary solvent delivery system, an on-line degasser, an autosampler, a column temperature controller, and a DAD (photo-diode-array-detector) detector. Agilent Zorbax SB-C18 column (5 μm, 4.6 × 250 mm) was employed during the experiment with a flow rate of 1.0 ml/min. The column temperature was maintained at 30 °C, and the injection volume was 10 μl. The mobile phase was composed of water (A) and acetonitrile (B) with the following gradient elution: 0–10 min, 95% A; 10–15 min, 95–90% A; 15–20 min, 90% A; 20–30 min, 90–85% A; 30–40 min, 85–80% A; 40–50 min, 80–75% A; 50-60 min, 75–70% A; 60-70 min, 70–0% A. The wavelength was set at 360 nm.

### Animals and BPH models

Male Spraue-Dawley rats (180–220 g) were procured from Shanghai Slac Laboratory Animal Co. Ltd. (Shanghai, China), and housed in the Laboratory Animal Center of Shanghai Jiao Tong University (Shanghai, China). The animals were housed in groups under controlled room temperature (25 ± 2 °C, 55 ± 10% relative humidity) with a 12/12 h light/dark cycle. Standard laboratory chow and water were available ad libitum. All experimental procedures were approved by the Animal Ethics Committee of Shanghai Jiao Tong University (Shanghai, China). All methods are reported in accordance with ARRIVE guidelines (https://arriveguidelines.org) for the reporting of animal experiments.

Following 1 week acclimation, rats were randomly assigned to 8 groups (*n* = 10) as following, Sham, BPH model, Finasteride, Pule’an, Low QCJ, High QCJ, Low Fr. B, High Fr. B. The scrotum of the sham animals were cut following sewing up without cutting off the both testicles. Rats in the other groups were castrated. After incision disinfection with penicillin for 1 week, sham rats were treated with saline (s.c., 0.5 ml/kg, alternate days) and 0.5% CMC-Na (i.g. 10 ml/kg, daily). BPH group rats were received testosterone propionate (s.c., 10 mg/kg) alternate days and 0.5% CMC-Na (i.g. 10 ml/kg) daily for 4 weeks [17, 18]. Two positive groups, the rats were treated with testosterone propionate (s.c., 10 mg/kg) and received a treatment with finasteride (i.g., 1 mg/kg) or Pule’an (i.g., 460 mg/kg). Low/High QCJ group rats were treated with testosterone propionate (s.c., 10 mg/kg) and received a treatment with QCJ (i.g., 860 mg/kg or 2580 mg/kg respectively). Low/High Fr. B group rats were treated with testosterone propionate (s.c., 10 mg/kg) and received a treatment with Fr. B (i.g., 160 mg/kg or 480 mg/kg respectively). Low dosage of QCJ or Fr. B was clinically equivalent doses (1.8 g Crude drug/kg). High dosage of QCJ or Fr. B was three times the clinical equivalent doses (5.4 g Crude drug/kg).

At the end of experiment, animals were sacrificed under anaesthesia after blood sample collection. The prostates were removed and ventral prostate tissues were fixed in 10% neutral buffered formalin and embedded in paraffin for both histological and immunohistochemical examinations. The remainder of each prostate was stored at − 80 °C and used for further analyses.

### Histology and immunohistochemical localizatiion of vascular endothelial growth factor (VEGF)

Prostate tissue were fixed for 1 day in paraformaldehyde solution (4% in phosphate-buffered saline (PBS) 0.1 M) at room temperature, dehydrated by graded ethanol and embedded in paraffin. Ten micrometres thick sections collected on glass slides, deparaffinized then stained with hematoxylin and eosin (H&E) for histopathological examination using light microscopy (Olympus BX51, Japan) associated to an Imaging system (Image Pro Plus 6.0).

Slices were deparaffinized and immersed in freshly prepared 3% H_2_O_2_, blocked with goat serum for 30 min, then treated with citrate buffer. The sections were rinsed with PBS and incubated at 4 °C overnight with VEGF antibody (Boster Biological Technology, Wuhan, China.), incubated with secondary antibody (goat anti-Rabbit IgG, Boster Biological Technology, Wuhan, China.) at 37 °C for 30 min. After washed with PBS, the sections were incubated in strept avidin-biotin complex at 37 °C for 30 min, and immersed in diaminobenzidine for 5 min. The haematoxylin-stained sections were dehydrated with ethanol and visualized with an optical microscope (Olympus BX51, Japan). The VEGF content was expressed as average optical density (IOD/Area).

### Evaluation of dihydrotestosterone (DHT), testosterone (T), estradiol (E2), tumor necrosis factor-α (TNF-α), interleukin-6 (IL-6) in plasma

DHT, T and E_2_ levels of the rats plasma were assayed with the commercially available kits (Shanghai Enzyme-linked Biotechnology Co., Ltd., Shanghai, China). TNF-α, IL-6 levels were assayed in plasma sample with the commercially available kits (MultiSciences Biotech Co., Ltd., Hangzhou, China). All the procedures were performed according to manufacturer’ s instructions of the kits.

### Evaluation of malondialdehyde (MDA), superoxide dismutase (SOD), glutathione peroxidase (GSH-Px), catalase (CAT) in prostate tissue

Prostate samples were homogenized and assayed for MDA, SOD, GSH-Px and CAT using commercially available kits (MultiSciences Biotech Co., Ltd., Hangzhou, China). All the procedures were performed according to manufacturer’s instructions of the kits.

### Statistical analysis

All data were expressed as mean ± standard deviation. The results were analyzed by one-way ANOVA followed by LSD for multiple comparisons using SPSS 21.0 software for Windows (SPSS Inc., Chicago, IL). A *P* value of < 0.05 was considered significant.

## Results

### Chemical ingredients of Fr. B

**Fig. 1:**
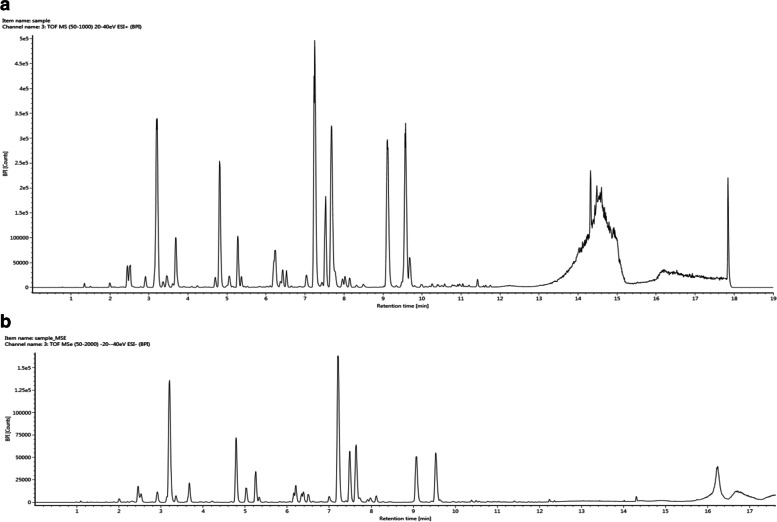
The BPI chromatograms of Fr. B by UPLC-QTOF-MS. BPI chromatogram in positive ESI mode; **b** BPI chromatogram in negative ESI mode

The BPI chromatograms of Fr. B by UPLC-QTOF-MS with both positive and negative ions were shown in Fig. 1. Ten flavonoid glycosides were assigned from Fr. B by their tandem mass data analysis of each peak in detail. Detailed information including retention time, accurate MS, and MS/MS fragmentions are listed in Table 1. Further, quantification of the 10 flavanoids glycosides in Fr. B was performed by HPLC with quercetin as internal standard, and compound 2 as index. The results showed that 10 flavonoid glycosides were accounted for 54.96% of all substance of Fr. B, in which compound 1 was with a relative content of 11.1%, and compound 2 was with a relative content of 13% in Fr. B.

**Table 1:** Flavonoid glycosides profile of Fr. B as characterized by UPLC-QTOF-MS analysis in MS^E^ mode

peak	t_R_(min)	formula	MW	[M + H]^+^	MS/MS	[M-H]^−^	MS/MS	Putative identity	reference
1	2.46	C_39_H_50_O_25_	918.2641	919.2706	773, 611, 465, 303	917.2817	755, 609, 463, 462, 301, 300	Quercetin 3-O-deoxyhexosyl-(1 → 6)-[deoxyhexosyl -(1 → 2)]-hexoside-7-O-hexoside	[19]
2	3.20	C_39_H_50_O_24_	902.2692	903.2755	757, 595, 449, 287	901.2599	739, 593, 447, 446, 285, 284	Kaempferol 3-O-deoxyhexosyl-(1 → 6)-[deoxyhexosyl -(1 → 2)]- hexoside-7-O-hexoside **(compound 1)**	[19]
3	3.67	C_33_H_40_O_21_	772.2062	773.2111	627, 465, 303	771.1976	609, 463, 301	Quercetin 3-O-deoxyhexosyl -(1 → 6)-hexoside − 7-O-hexoside	[19]
4	4.78	C_33_H_40_O_20_	756.2113	757.2175	611, 449, 287, 228	755.2016	593, 447, 285	Kaempferol 3-O-deoxyhexosyl -(1 → 6)-hexoside − 7-hexoside	[20]
5	5.25	C_34_H_42_O_21_	786.2219	787.2267	641, 479, 317	785.2145	623, 477, 315	Isorhamnetin 3-O- deoxyhexosyl -(1 → 6)-hexoside − 7-O-hexoside	[19]
6	7.21	C_33_H_40_O_19_	740.2164	741.2215	595, 449, 287	739.2073	593, 285, 284	Kaempferol 3-O-deoxyhexosyl -(1 → 6)-[deoxyhexosyl-(1 → 2)]-hexoside **(compound 2)**	[19]
7	7.48	C_34_H_42_O_20_	770.2269	771.2328	625, 479, 317	769.2176	623, 315, 314	3-O- deoxyhexosyl -(1 → 6)-[deoxyhexosyl -(1 → 2)]- hexoside	[19]
8	7.64	C_27_H_30_O_16_	610.1534	611.1610	465, 303	609.1450	463, 301, 300	Quercetin 3-O-deoxyhexosyl -(1 → 6)-hexoside	[19]
9	9.08	C_27_H_30_O_15_	594.1585	595.1665	449, 287	593.1495	447, 285	Kaempferol 3-O-deoxyhexosyl -(1 → 6)-hexoside	[19]
10	9.53	C_28_H_32_O_16_	624.169	625.1756	479, 317	623.1609	477, 315	Isorhamnetin 3-O-deoxyhexosyl -(1 → 6)-hexoside	[19]

Compounds 1 and 2 were further determined by reference compounds

### Effect of Fr. B on prostate morphology of BPH rats

**Fig. 2:**
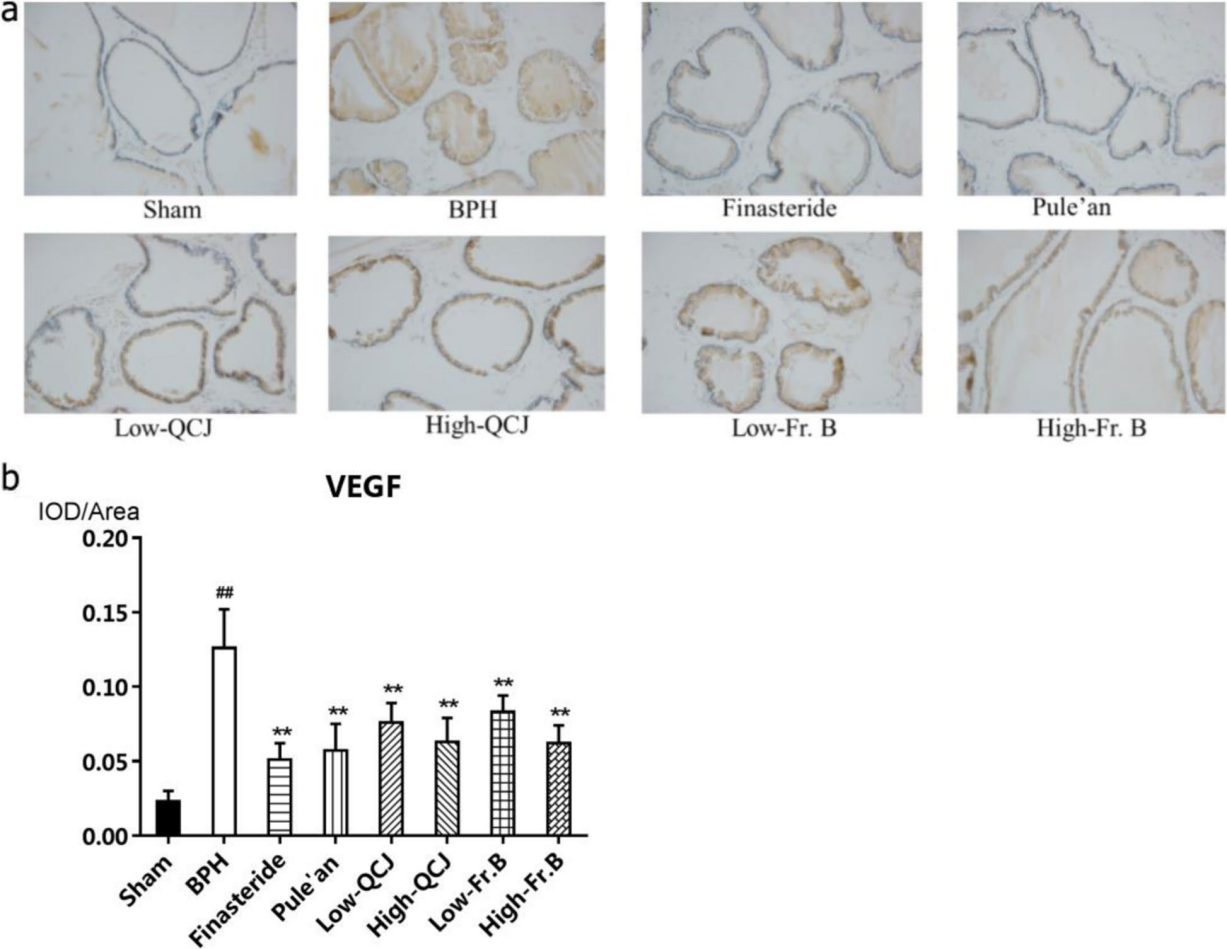
Effects of QCJ and Fr. B onprostatic histopathology morphology (**a**) and the expression of VEGF (**b**) in BPH rats. ^##^*P* < 0.01, versus Sham group; ^*^P < 0.05, ^**^P < 0.01, versus BPH group

There was no significant difference in rat weight among the groups along with the experiment. Prostate tissue collected from the sham rats showed normal architecture and histology, regular acini with cuboidal and low cylindrical epithelium with round nuclei showing basal alignment. While a significant disorganization of prostate tissue, irregular acinar shape with papillary projection into the lumen and foci of piling-up hyperplastic nodules were observed in the prostate tissue of rats after BPH induction. Finasteride, Pule’an, QCJ and Fr. B treatment could significantly improved the histological pattern and marked hyperplasia of prostate tissuein BPH rats (Fig. 2a). The expression of VEGF in the rat prostates was analyzed by immunohistochemical analysis. As shown in Fig. 2b, compared with the sham operation group, VEGF level in the prostate tissue of BPH group rats was significantly increased (*P* < 0.01). Finasteride, Pule’an, QCJ and Fr. B significantly reduced the expression of VEGF in the prostate of BPH rats (*P* < 0.01), and the high dose of QCJ and Fr. B decreased the VEGF level by 50.04 and 51.02%, while low dose decreased the level of VEGF by 39.67 and 34.36% respectively, which indicated that QCJ and Fr. B might regulate the expression of VEGF to reduce the abnormal proliferation of prostate cells and alleviate BPH.

### Effects of Fr. B on sex hormone levels in BPH rats serum

**Fig. 3:**
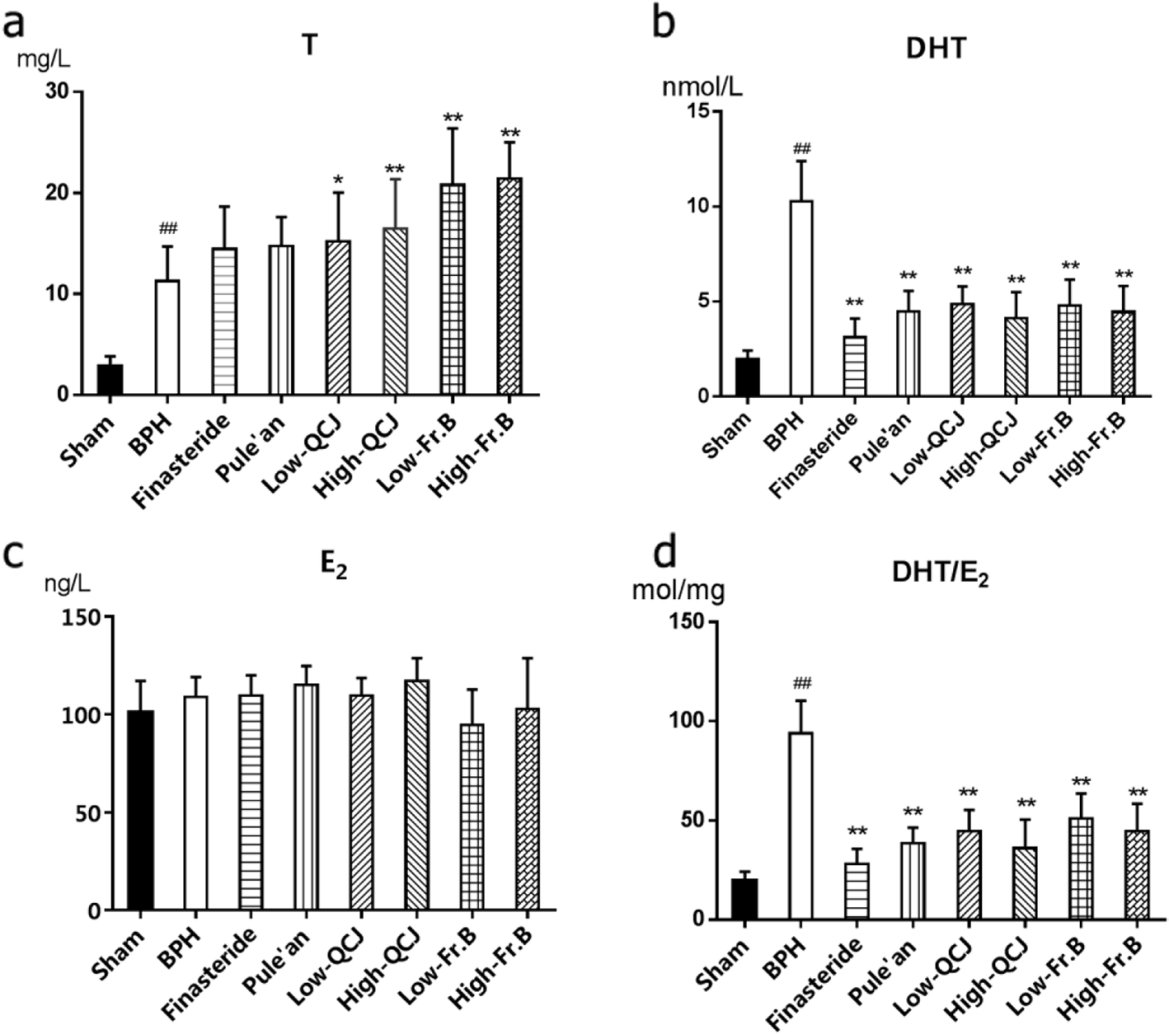
Effects of QCJ and Fr. B on the levels of DHT (**a**), T (**b**), E_2 _(**c**) and DHT/E_2 _(**d**) in serum of BPH rats. ^##^*P* < 0.01, versus Sham group; ^*^P < 0.05, ^**^P < 0.01, versus BPH group

**Fig. 4:**
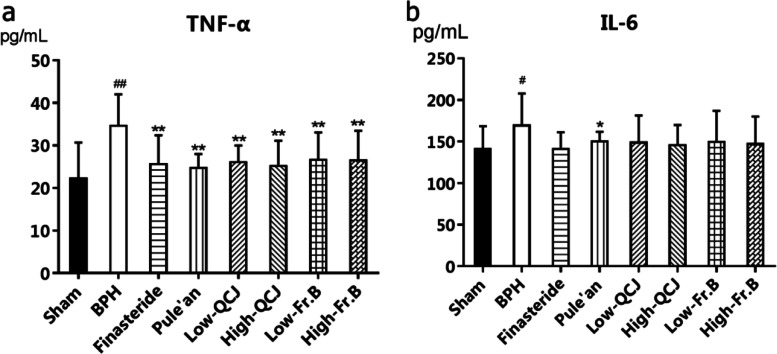
Effects of QCJ and Fr. B on the levels of TNF-α (**a**) and IL-6 (**b**) in serum of BPH rats. ^#^*P* < 0.05, ^##^*P* < 0.01versus Sham group; ^*^P < 0.05, ^**^P < 0.01, versus BPH group

The basal levels of DHT, T and E_2_ in serum of sham animals were showed in Fig. 3, those in serum collected from BPH animals showed marked increases (*P* < 0.01). Treatment with finasteride, Pule’an, QCJ and Fr. B considerably reduced DHT and DHT/E_2_ levels (*P* < 0.01). However, significant increases were observed in T levels with QCJ and Fr. B administration (*P* < 0.05, Fig. 4). DHT is converted by the steroid enzyme 5α-reductase from T [21]. The production and accumulation of DHT in the prostate promote cell growth and induce hyperplasia [22]. The level of DHT in serum of rats was significantly lower than that of BPH rats, while the level of T was significantly higher, suggesting that Fr. B may play an anti-prostate hyperplasia role by inhibiting the production of DHT.

### Effects of Fr. B on inflammatory factors in BPH rats serum

As shown in Fig. 4, serum levels of TNF-α and IL-6 were markedly elevated in BPH rats (*P* < 0.05). Oral administration of finasteride, Pule’an, QCJ and Fr. B to BPH rats considerably reduced TNF-α levels (*P* < 0.01). Pule’an could significantly decrease IL-6 levels of BPH rats (*P* < 0.05). However, finasteride, QCJ and Fr. B groups showed decreasing trends in IL-6 levels without significant effect statistically (Fig. 4).

### Effects of Fr. B on MDA, SOD, GSH-Px and CAT levels in BPH rats prostates tissues

**Fig. 5:**
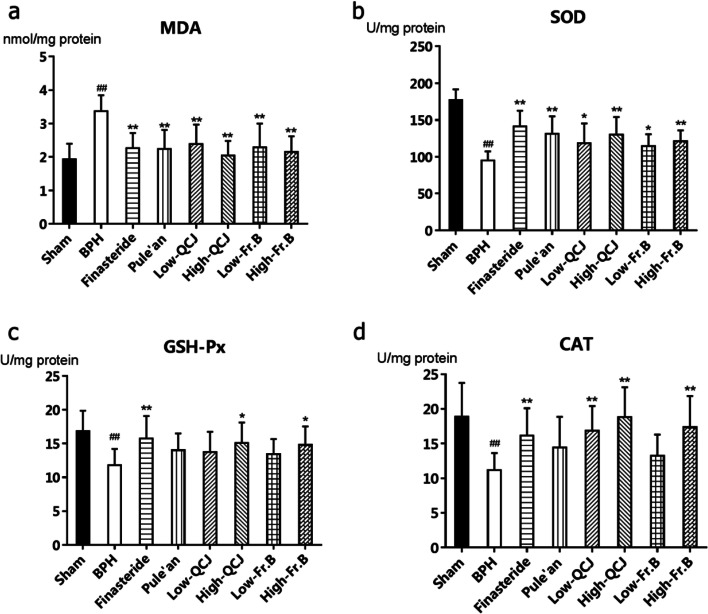
Effects of QCJ and Fr. B on oxidative stress parameters in prostate tissue of rats. **a** MDA; **b** SOD; **c** GSH-Px; **d** CAT; ^##^*P* < 0.01, versus Sham group; ^*^P < 0.05, ^**^P < 0.01, versus BPH group

As shown in Fig. 5, homogenates of the prostates tissues from sham animals had a basal levels of MDA, while that from BPH rats showed a marked increase (*P* < 0.01). Oral administration of finasteride, Pule’an, QCJ and Fr. B to BPH rats significantly decreased levels of MDA (*P* < 0.01). Moreover, we also observed that the contents of SOD, GSH-Px and CAT in BPH rats prostates were significantly reduced, these are three important endogenous antioxidants in the body. Administration of finasteride, Pule’an, QCJ and Fr. B to BPH rats could significantly increase SOD levels of BPH rats (*P* < 0.05). Similarly, treatment with finasteride, high dose of QCJ and Fr. B also demonstrated significant trends of protection increasing the reduced levels of CAT and GSH-Px (*P* < 0.05) as well, whereas Pule’an group showed an increasing trend without statistically significance.

## Discussion

Phytotherapy has been playing an important role in the treatment of BPH for over decades because of its mildness, effectiveness and low adverse effects. Lots of the researchers revealed that several kinds of constituents including the fatty acids, polyphenols, flavonoids, phytosterols, alkanoids may be responsible for BPH inhibitory activities of phytotherapy and the suggested mechanisms includes 5α-reductase inhibitor, α1-adrenoceptor antagonist, aromatase inhibitor, anti-androgen, growth factor inhibitor, and so on [23–25]. The special chemical structure of flavonoids makes them have good anti-BPH properties [26]. Total flavanol glycosides from *Abacopteris penangiana* and its acid hydrolysate [27], total flavonoid extract of *Pteris multifida* [15] and dihydroquercetin [28], were reported to exert BPH protective effect through regulating inflammatory responses and reducing oxidative stress. In this study, we found that the active fraction Fr. B of *P. utilis* leaves riched in flavanoids, of which 10 identified flavonoid glycosides accounted for 54.96% of all Fr. B substances, which contributed to BPH reduction by mechanisms related with anti-oxidant, anti-inflammatory effect, reduction of DHT/E2 ratio, and inhibition of growth factor. Among these 10 identified flavonoid glycosides, 4 compounds are kaempferol-*O*- glycosides (peak 2, 4, 6, 9), 3 compounds are quercetin-*O*-glycosides (peak 1, 3, 8), and 3 compounds are isorhamnetin-*O*-glycosides (peak 5, 7, 10). It was reported that kaempferol exhibited its androgenic-like activity and served as a selective androgen receptor modulator that contributed to androgen-related BPH development [29]. Kaempferol was revealed to be much better inhibitor of the type 2 than type 1 isozyme, quercetin was proved to be a potent inhibitor of the type 1 5α-reductase [30]. Moreover, kaempferol and its glycosides (kaempferol3-(3-*E*-*p*-coumaroyl-α-L-rhamnopyranoside), kaempferol 3-(2,3-*di*-*E*-*p*-coumaroyl-α-L-rhamnopyranoside)) from the pollen of *Brassica napus* L. were found to exhibit down-regulation of prostate specific antigen in LNCaP cells [31]. Yin et al [32] once reported the kaempferol-3-*O*-α-L-rhamnopyranosyl-(1 → 6)-β-D-glucopyranoside isolated from the *Adina rubella* leaves, the extract of which exhibited anti-oxidative and anti-inflammatory activities and 5α-reductase inhibition associated with BPH. Similarly, *Epilobium angustifolium* exhibited the therapeutic potential against BPH, and its active compounds included both kaempferol and quercetin as well as their glycosides [33]. The chemical investigation of the aerial parts of the fern *Asplenium ceterach*, one traditional medicine to treat BPH, revealed the presence of kaempferol and quercetin glycosides [34]. In addition, two quercetin glycosides (quercetin5-*O*-β-D-glucopyranoside, quercetin 3-*O*-β-L-rhamno-pyranoside) are the main active components against anti-BPH of aqueous extracts from *Saxifraga stolonifera* [35]. So, the flavonoid glycosides identified from Fr. B should be potential anti-BPH ingredients of *P. utilis*, for further purification and pharmacological investigation.

In vitro and in vivo studies describe oxidative stress as a major pathway involved in the occurrence of BPH [36, 37]. High plasma peroxide levels were found in BPH patients compared with controls [38, 39]. Circulating MDA levels were found to be significantly higher in BPH patients than in healthy donors [38]. However, other works found circulating MDA levels in BPH patients similar to those in controls [36]. In our study, the level of MDA in prostate of BPH rats was significantly increased, indicating the prostate tissue was in a state of oxidative damage. Fr. B could significantly decrease the level of MDA in prostate of BPH rats. Normally, highly oxidative stresses are removed by natural protective mechanism, the superoxide dismutase enzyme system, such as SOD, GSH-Px and CAT [17, 40, 41]. Fr. B could significantly increase the content of antioxidant enzymes (SOD, GSH-Px and CAT) and enhance the antioxidant capacity of rats. Prostate enlargement due to chronic inflammatory process may progressively conduce to BPH progression. Therefore, inflammation is a therapeutic target for BPH [42]. Although Fr. B had no significant decrease in serum IL-6 in BPH rats, it had a significant effect on TNF-α, suggesting its potential to improve BPH-related inflammation. These results suggested that Fr. B attenuate symptoms of BPH, at least in part, by decreasing the proinflammatory cytokines secretion and oxidative stress.

In addition, androgens are essential for the development and differentiated function of the prostate, as well as for proliferation and survival of prostatic cells [43, 44]. It is clear that androgens, estrogens [45] and growth factors [46] contribute to the BPH, but the exact etiology remains unknown. In this study, increased DHT levels and decreased T levels, as well as increased DHT/E_2_ resulting from BPH induction, were significantly improved by Fr. B treatment. This reflected the androgens regulation potential mechanism of Fr. B on treating BPH. Recently, increasing evidences suggested that prostate growth was under the indirect control of androgens through the mediation of different growth factors [47]. VEGF is a major inducer of angiogenesis as it influences endothelial cell growth. The immunohistochemical assay showed that the expression of VEGF in the prostatic tissue with BPH rats were significantly increased compared normal prostatic tissue [48]. We also found that Fr. B could significantly decrease the expression of VEGF in prostate tissue of BPH rats. Its specific mechanism and signal pathway need to be further discussed.

## Conclusion

Collectively, the results of present study showed that Fr. B prepared from the water extract of *P. utilis* leaves attenuated symptoms of BPH through multiple mechanisms including reduction of DHT/E2 ratio, inhibition of growth factor, anti-inflammation and anti-oxidation. Ten flavonoids were assigned from Fr. B by UPLC-QTOF-MS. These results support that Fr. B should be further explored as a prospective natural foods or supplements for BPH treatment.

## Data Availability

All data analysed during this study are included in this published article. The datasets generated during this study are not publicly available but are available from the corresponding author on reasonable request.

## Abbreviations

*P. utilis*: *Prinsepia utilis* Royle
BPH: Benign prostatic hyperplasia
QCJ: Water extract from *P. utilis* leaves
Fr. B: Fraction separated from QCJ
UPLC-QTOF-MS: Ultra performance liquid chromatography-quadrupole-time of flight-mass spectrometry
HPLC: High-performance liquid chromatography
DAD: Photo-diode-array-detector
ESI: Electrospray ionization
MSE: Mass spectrometry elevated energy
VEGF: Vascular endothelial growth factor
DHT: Dihydrotestosterone
T: Testosterone
E2: Estradiol
TNF-α: Tumor necrosis factor-α
IL-6: Interleukin-6
MDA: Malondialdehyde
SOD: Superoxide dismutase
GSH-Px: Glutathione peroxidase
CAT: Catalase
